# Patient-Reported Tolerance of Magnetic Resonance-Guided Radiation Therapy

**DOI:** 10.3389/fonc.2020.01782

**Published:** 2020-09-21

**Authors:** Mutlay Sayan, Ilkay Serbez, Bilgehan Teymur, Gokhan Gur, Teuta Zoto Mustafayev, Gorkem Gungor, Banu Atalar, Enis Ozyar

**Affiliations:** ^1^Department of Radiation Oncology, Rutgers Cancer Institute of New Jersey, New Brunswick, NJ, United States; ^2^Department of Radiation Oncology, School of Medicine, Mehmet Ali Aydınlar Acıbadem University, Istanbul, Turkey

**Keywords:** MR-guided, MR-linac, patient tolerance, patient-reported outcomes, radiotherapy

## Abstract

**Purpose:**

Magnetic resonance-guided radiation therapy (MRgRT) has been incorporated into a growing number of clinical practices world-wide, however, there is limited data on patient experiences with MRgRT. The purpose of this study was to prospectively evaluate patient tolerance of MRgRT using patient reported outcome questionnaires (PRO-Q).

**Methods:**

Ninety patients were enrolled in this prospective observational study and treated with MRgRT (MRIdian Linac System, ViewRay Inc. Oakwood Village, OH, United States) between September 2018 and September 2019. Breath-hold-gated dose delivery with audiovisual feedback was completed as needed. Patients completed an in-house developed PRO-Q after the first and last fraction of MRgRT.

**Results:**

The most commonly treated anatomic sites were the abdomen (47%) and pelvis (33%). Respiratory gating was utilized in 62% of the patients. Patients rated their experience as positive or at least tolerable with mean scores of 1.0–2.8. The most common complaint was the temperature in the room (61%) followed by paresthesias (57%). The degree of anxiety reported by 45% of the patients significantly decreased at the completion of treatment (mean score 1.54 vs. 1.36, *p* = 0.01). Forty-three percent of the patients reported some degree of disturbing noise which was improved considerably by use of music. All patients appreciated their active role during the treatment.

**Conclusion:**

This evaluation of PROs indicates that MRgRT was well-tolerated by our patients. Patients’ experience may further improve with adjustment of room temperature and noise reduction.

## Introduction

Image-guided radiation therapy (IGRT) is a sub-set of the motion management strategies clinically implemented to help mitigate motion-related errors. Conventional IGRT techniques include kilovoltage or megavoltage computed tomography (CT) imaging (1). However, onboard CT imaging not only offers a poor soft tissue contrast but also results in additional undesirable radiation exposure.

Magnetic resonance imaging (MRI) provides better soft tissue contrast than CT imaging and does not expose patients to additional imaging dose. Integrating an MRI scanner into a radiation therapy delivery system enhances the delineation of tumor and organs-at-risk (OAR), accurate patient setup and adaptation of the treatment to interfractional anatomy changes (2, 3). MR-guided radiation therapy (MRgRT) also enables monitoring of intrafractional motion and real-time adaptation of the treatment delivery (4–6). Furthermore, utilization of video feedback to patients during the delivery of gated MRgRT allows them to have an active role in their treatment.

Magnetic resonance-guided radiation therapy has been successfully incorporated into a growing number of clinical practices world-wide (7). While the feasibility of MRgRT has been reported in prior studies, data on patient reported outcomes are limited. In this study, we prospectively evaluate the patient tolerance of MRgRT using a patient reported outcome questionnaires (PRO-Q).

## Materials and Methods

All patients receiving MRgRT enrolled in this prospective observational study at Acibadem Maslak Hospital between September 2018 and September 2019. This study was approved by the institutional review board of Acibadem Mehmet Ali Aydinlar University (IRB: 2018-16/2). MRIdian Linac system (ViewRay Inc., Oakwood Village, OH, United States) was utilized for simulation and treatment delivery. It consists of a split, super-conductor low-field (0.35 tesla) MRI system with a 70-cm bore and a 50-cm field-of-view. The RT delivery system includes a ring-gantry mounted 6 MV linear accelerator and double-focused multi-leaf collimator.

**FIGURE 1:**
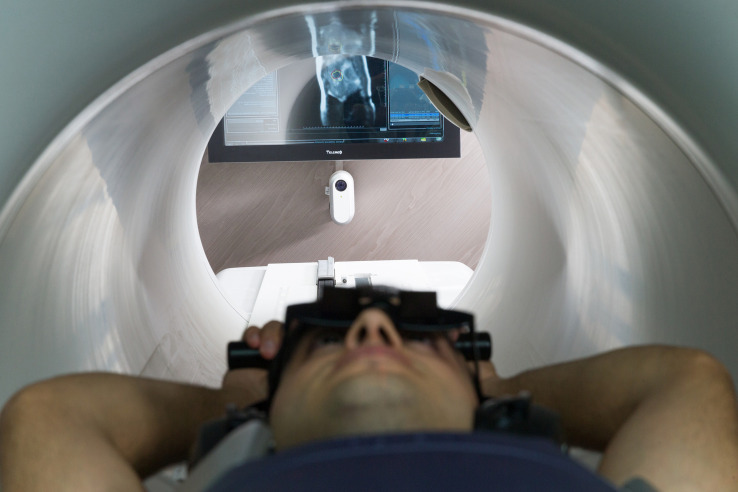
Patient set-up during magnetic resonance-guided radiation therapy.

In order to minimize the respiratory movement when needed, prismatic glasses were utilized for patients to see the colored gating target and the gating boundary contours on an MR-compatible monitor just posterior to the patient and the machine (Figure 1). A short low-resolution scan was first performed to correct the position of the patient. A high-resolution scan was then performed to detect inter-fractional daily anatomical changes of the tumor site and OARs for further contouring and adaptive planning. Following simulation, target volumes and OARs were delineated and a DVH was generated. After the plan was finalized, treatment was started and on-time cMRI was utilized for 2D-tracking on sagittal plane. Re-planning and plan comparison were performed on subsequent days to optimize the plan. The temperature in the treatment room was kept at 20°C.

Patient-reported tolerance of the entire treatment procedure was recorded using an in-house developed PRO-Q (Table 1). The PRO-Q was derived from published studies and consists of questions relevant to the MRgRT (8–12). The questionnaire was completed after the first and last fraction of MRgRT. The questionnaire consisted of questions regarding potential MR-related experiences and complaints (e.g., noise, bore size, fixation with coils). Items were scored using a four-point scale. A score below 3 is considered to be tolerable in the absence of treatment break or termination. Statistical analyses were performed using SPSS statistical software version 25 (IBM Corp., Armonk, NY, United States). The Wilcoxon signed rank test was used to compare matched samples. A *p*-value < 0.05 was considered statistically significant.

**TABLE 1:** Patient-reported outcome (PRO) questionnaire.

How do you rate…		1 2 3 4	
… your anxiety level during treatment?	Not at all		Very much
… the duration of treatment?	Very short		Very long
… the sensation of local heat?	Not at all		Very warm
… the feeling of cold during treatment?	Not at all		Very cold
… dizziness?	Not at all		Very much
… potential tingling sensations in your extremities	Not at all		Very much
… a metallic taste?	Not at all		Very much
… perceptions of light flashes?	Not at all		Very much
… the noise in the MRI?	Very quiet		Very loud
Was music relaxing?	Not at all		Very much
Was it difficult to control the target by holding your breath?	Not at all		Very much
Was it disturbing to see your tumor during treatment?	Not at all		Very much
Did you like having an active role during treatment?	Not at all		Very much
Did you worry about your contribution to the treatment?	Not at all		Very much

## Results

Ninety patients were enrolled and treated with MRgRT during the study period. Baseline patient characteristics are shown in Table 2. Patients had a median age of 66 (range, 23–85). Sixty-seven percent of the patients were male. The most frequently treated anatomic site was the abdomen (47%) followed by the pelvis (33%). Stereotactic body radiotherapy (SBRT) was delivered in 81% of the patients. The mean prescription dose was 43.34 Gy (range, 24–70 Gy). The median number of fractions per patient was 5 (range, 3–28). Total number of fractions delivered was 560. Respiratory gating was utilized in 62% of the patients. Median treatment delivery time was 45 min (range of 42–64 min).

**TABLE 2:** Demographic and clinical characteristics of the patients.

Patient, *n*	90
Age, years, median (range)	66 (23–85)
**Gender, *n* (%)**	
Female	27 (30)
Male	63 (70)
**Anatomic site treated, *n* (%)**	
Abdomen	42 (47)
Pelvis	30 (33)
Thorax	18 (20)
**Radiation therapy**	
Dose (Gy), mean, range	40 (24–70)
Number of fractions, median, range	5 (3–28)
Breath-hold, *n* (%)	56 (62)

All patients completed the questionnaires after the first and last fraction of the treatment. Overall, patients rated their experience as positive or at least tolerable with mean scores of 1.0–2.8 (Table 3). Forty-five percent of the patients reported some degree of anxiety after the first fraction (mean score 1.54), however this was significantly decreased at the end of treatment (mean score 1.36) (*p* = 0.01). Otherwise, there were no statistically significant changes between the first fraction and at the end of treatment for the rest of the assessed questions.

**TABLE 3:** Result of the patient reported outcomes.

How do you rate…	After the first fraction, Mean (SD)	After the last fraction, Mean (SD)	*p*
… your anxiety level during treatment?	1.44 (0.656)	1.26 (0.567)	0.01
… the duration of treatment?	2.73 (0.747)	2.80 (0.741)	0.38
… the sensation of local heat?	1.14 (0.436)	1.12 (0.364)	0.63
… the feeling of cold during treatment?	1.83 (0.604	1.74 (0.728)	0.19
… dizziness?	1.63 (0.661)	1.53 (0.640)	0.09
… potential tingling sensations in your extremities	1.78 (0.790)	1.70 (0.729)	0.22
… a metallic taste?	1.03 (0.184)	1.07 (0.252)	0.32
… perceptions of light flashes?	1.04 (0.207)	1.04 (0.208)	0.99
… the noise in the MRI?	1.66 (0.823)	1.53 (0.694)	0.26
Was music relaxing?	3.21 (1.258)	3.10 (1.274)	0.22
Was it difficult to control the target by holding your breath?	2.00 (0.788)	1.86 (0.805)	0.21
Was it disturbing to see your tumor during treatment?	1.34 (0.745)	1.25 (0.606)	0.35
Did you like having an active role during treatment?	2.96 (0.852)	3.11 (0.867)	0.15
Did you worry about your contribution to the treatment?	1.32 (0.640)	1.22 (0.623)	0.20

Sixty-eight percent of patients reported at least some degree of potential MR – related complaints. The most common complaint was the temperature in the room (61%) followed by paresthesias (57%). Furthermore, 43% of the patients reported experiencing disturbing noise. Music was requested by 28 patients during the treatment and of those participants, 78% found music to be relaxing. No patients reported severe difficulties to control the target during breath-hold delivery. Only two patients reported that seeing their tumor during treatment was disturbing to them. All patients appreciated their active role during the treatment.

## Discussion

Patient-reported outcomes are important tools to assess the quality of life and tolerability of the treatment. We previously reported that MRgRT has been successfully implemented into clinical practice at our institution (13). In this evaluation of PROs, we showed that MRgRT is well-tolerated by our patients with some degree of MR-related complaints.

The MR-guidance improves accuracy in treatment planning and safe delivery of EBRT. It enhances several aspects of EBRT workflow for most disease sites, including OAR delineation, patient setup, online motion monitoring and plan adaptations. Given the clinical advantages of MR-guidance, a number of institutions in Europe and the United States have successfully introduced the MRgRT into routine clinical practice in recent years (14–17). A number of clinical and technical challenges surrounding integration of MRgRT have been extensively discussed previously (18). However, it is imperative to additionally explore the potential challenges patients may experience with this new treatment modality.

In our cohort, all patients completed their treatment course with no unanticipated treatment breaks. Furthermore, evaluation of PROs in our study indicates that MRgRT is well-tolerated, findings which are in agreement with results published in two separate studies by Tetar et al. (9) and Kluter et al. (19). Tetar et al. (9) designed the first study evaluating the patient tolerance of MRgRT. In this study, an in-house developed PRO-Q was completed by 150 patients immediately following their last fraction of MRgRT. Their findings indicated that treatment was well-tolerated; only 5% of the patients reported the treatment duration to be unacceptably long. Kluter et al. (19) prospectively assessed the patient tolerance of MRgRT using an in-house developed PRO-Q. Forty-three patients completed PRO-Q after the first fraction, weekly during the treatment, and after the last fraction of MRgRT. Kluter et al. (19) also reported that the treatment course was generally well-tolerated by all patients in their study with no significant changes appreciated during the treatment course. While the rate of MR-related patient complaints in our study (68%) was high as compared to the rate reported (29%) by Tetar et al. (9), it was similar to the rate reported (65%) by Kluter et al. (19). The main concern in our cohort was the room temperature and paresthesias. We are now treating most of our patients in the arms-down position which is expected to increase patient comfort. None of our patients reported considerable treatment-related anxiety (score ≥3) and the average score declined significantly over the course of treatment (36% vs. 26%, *p* = 0.01). Use of music was relaxing for the majority of patients, particularly in those who reported noise disturbance with the sound generated by the magnetic resonance equipment.

The limitations of our study include its small sample size and inherent confounding factors, such as use of music, that cannot be completely accounted for in a non-randomized study. In addition, while our in-house developed PRO-Q is similar to questionnaires used in prior studies, it is not a validated tool and results may be influenced by patient’s own interpretations, background, and desirability of answer.

## Conclusion

Based on the evaluation of PRO-Qs, MRgRT was generally well-tolerated with no early termination of radiation treatment. Our results suggest that efforts to reduce noise disturbances and adjustment of room temperature may improve a patient’s experience during MRgRT. Larger prospective clinical studies with a validated questionnaire are warranted to further evaluate the patient experience during MRgRT.

## Data Availability Statement

All datasets presented in this study are included in the article/supplementary material.

## Ethics Statement

The studies involving human participants were reviewed and approved by the Mehmet Ali Aydınlar Acıbadem University. The patients/participants provided their written informed consent to participate in this study. Written informed consent was obtained from the individual for the publication of any potentially identifiable images.

## Author Contributions

All authors listed have made a substantial, direct and intellectual contribution to the work, and approved it for publication.

## Conflict of Interest

The authors declare that the research was conducted in the absence of any commercial or financial relationships that could be construed as a potential conflict of interest.
